# Supplementary material for: Usability and acceptance of crowd-based early warning of harmful algal blooms
Source: PeerJ. 2023 Mar 1;11:e14923. doi: 10.7717/peerj.14923 (PMC9985416; doi:10.7717/peerj.14923)
Supplement: Supplemental Information 1 [file peerj-11-14923-s001.pdf]

Appendix A. Kuesioner

Table A.1: Kuesioner "Acceptance"

Konstruk	Item	Kuesioner	Sangat tidak setuju (1)	Tidak setuju (2)	Netral/ tidak tahu (3)	Setuju (4)	Sangat setuju (5)
AWA	awa1	Saya menyadari potensi ancaman HABs					
	awa2	Saya memiliki pengetahuan yang cukup tentang akibat kejadian HABs					
	awa3	Saya terus memperbarui diri dalam hal kesadaran mengenai HABs					
	awa4	Saya berbagi informasi pengetahuan HABs kepada rekan-rekan saya untuk meningkatkan kesadaran saya					
PEU	peu1	Mempelajari penggunaan Alboom adalah hal yang mudah bagi saya					

	peu2	Mudah bagi saya untuk menjadi mahir dalam menggunakan Alboom					
	peu3	Penggunaan Alboom jelas dan dapat dimengerti					
	peu4	Secara keseluruhan mudah bagi saya untuk menggunakan Alboom					
PUF	puf1	Alboom memberikan informasi yang bermanfaat bagi saya					
	puf2	Alboom menambah wawasan saya terhadap upaya penanggulangan HABs					
	puf3	Menggunakan Alboom adalah hal yang relevan atau berguna bagi pekerjaan saya					
ATT	att1	Menurut saya, menggunakan Alboom adalah ide yang bagus					
	att2	Saya memiliki sikap positif terhadap penggunaan Alboom					

	att3	Dengan mempertimbangkan semua hal, penggunaan Alboom adalah hal yang disarankan					
	att4	Saya pikir menggunakan Alboom adalah hal yang menarik dan menyenangkan					
SOC	soc1	Saya perlu menggunakan Alboom menurut rekan-rekan saya					
	soc2	Menurut orang-orang yang mempengaruhi perilaku saya, saya perlu menggunakan Alboom					
	soc3	Jika masyarakat pesisir merasa terbantu dengan Alboom, maka saya perlu menggunakan aplikasi ini					
REW	rew1	Saya berharap menerima imbalan (dapat berupa sertifikat, kredit, ataupun lainnya) atas kontribusi saya ke dalam Alboom					

	rew2	Semakin banyak imbalan yang saya dapatkan, semakin saya ingin berkontribusi ke dalam Alboom					
	rew3	Saya merasa puas dengan penghargaan/reward yang diberikan dalam penggunaan Alboom					
INT	int1	Berdasarkan pengalaman saya, kemungkinan besar saya akan terus berkontribusi ke dalam Alboom					
	int2	Saya akan merekomendasikan orang lain untuk menggunakan Alboom					
	int3	Saya berencana untuk sering menggunakan Alboom di masa mendatang					

Table A.2: Kuesioner ”Usability”

**Kode**	**Kuesioner**	**Sangat tidak setuju (1)**	**Tidak setuju (2)**	**Netral/ tidak tahu (3)**	**Setuju (4)**	**Sangat setuju (5)**
sus1	Saya pikir saya akan menggunakan Alboom lagi					
sus2	Saya merasa Alboom rumit untuk digunakan					
sus3	Saya merasa Alboom mudah digunakan					
sus4	Saya membutuhkan bantuan dari orang lain atau teknisi dalam menggunakan Alboom					
sus5	Saya merasa fitur-fitur Alboom berjalan dengan semestinya					
sus6	Saya merasa ada banyak hal yang tidak konsisten (tidak serasi) pada Alboom					
sus7	Saya merasa orang lain akan memahami cara menggunakan Alboom dengan cepat					
sus8	Saya merasa Alboom membingungkan					
sus9	Saya merasa tidak ada hambatan dalam menggunakan Alboom					
sus10	Saya perlu membiasakan diri terlebih dahulu sebelum menggunakan Alboom					

Appendix A. Questionnaires

Table A.1: Acceptance questionnaires

Construct	Item	Questionnaire	Strongly disagree (1)	Disagree (2)	Neutral (3)	Agree (4)	Strongly agree (5)
AWA	awa1	I am aware of the potential threat of HABs					
	awa2	I have sufficient knowledge about the consequences of the HABs incident					
	awa3	I keep renewing myself in awareness of HABs					
	awa4	I share HABs knowledge with my colleagues to increase awareness					
PEU	peu1	Learning to use Alboom is easy for me					
	peu2	It is easy for me to become proficient in using Alboom					
	peu3	The use of Alboom is clear and easy to understand					
	peu4	Overall, it is easy for me to use Alboom					

PUF	puf1	Alboom provides useful information to me					
	puf2	Alboom adds to my knowledge about HABs prevention efforts					
	puf3	Using Alboom is relevant or useful for my work					
ATT	att1	I think using Alboom is a good idea					
	att2	I have a positive attitude towards using Alboom					
	att3	All things considered, the use of Alboom is recommended					
	att4	I think using Alboom is interesting and fun					
SOC	soc1	I need to use Alboom according to my colleagues' opinions					
	soc2	According to people who influence my behavior, I must use Alboom					
	soc3	If coastal communities feel helped by Alboom, then I must use this application					

REW	rew1	I hope to receive a reward (e.g., a certificate, credit or otherwise) for my contribution to Alboom					
	rew2	The more rewards I get, the more I want to contribute to Alboom					
	rew3	I am satisfied with the rewards given in using Alboom					
INT	int1	Based on my experience, I will most likely continue to contribute to Alboom					
	int2	I will recommend others to use Alboom					
	int3	I plan to use Alboom often in the future					

Table A.2: Usability questionnaires

**Code**	**Questionnaire**	**Strongly disagree (1)**	**Disagree (2)**	**Neutral (3)**	**Agree (4)**	**Strongly agree (5)**
sus1	I think that I would like to use Alboom					
sus2	I found Alboom unnecessarily complex					
sus3	I thought Alboom was easy to use					
sus4	I think that I would need the support of a technical person to use Alboom					
sus5	I found the various functions in Alboom were well integrated					
sus6	I thought there was markedly well inconsistency in Alboom					
sus7	I would imagine that most people would learn to use Alboom very quickly					
sus8	I found Alboom very cumbersome to use					
sus9	I felt very confident using Alboom					
sus10	I required to learn a lot of things before I could get going with Alboom					
